# Reduced physical activity in children and adolescents with Juvenile Idiopathic Arthritis despite satisfactory control of inflammation

**DOI:** 10.1186/s12969-015-0053-5

**Published:** 2015-12-10

**Authors:** Anna-Helene Bohr, Susan Nielsen, Klaus Müller, Freddy Karup Pedersen, Lars Bo Andersen

**Affiliations:** Department of Paediatrics and Adolescent Medicine, JMC Research Unit, Rigshospitalet, Afs. 7821, Tagensvej 22, Copenhagen N, Denmark; Department of Paediatrics and Adolescent Medicine and Institute for Inflammation Research, Rigshospitalet, Copenhagen N, Denmark; Department of Sports Sciences and Clinical Biomechanics, University of Southern Denmark, Odense, Denmark; Norwegian School of Sport Science, Oslo, Norway

**Keywords:** Juvenile Idiopathic Arthritis, Chronic arthritis, Disease activity, Physical activity, Premature atherosclerosis

## Abstract

**Background:**

Vascular health is of concern in patients with Juvenile Idiopathic Arthritis (JIA) since Rheumatoid Arthritis (RA) epidemiologically has a well-described association with premature development of atherosclerosis. Chronic inflammation with persisting systemic circulating inflammatory proteins may be a cause of vascular damage, but general physical inactivity could be an important contributor. Pain and fatigue are common complaints in patients with JIA and may well lead to an inactive sedentary lifestyle. For this reason we assessed the physical activity (PA) objectively in patients with moderate to severe Juvenile Idiopathic Arthritis (JIA) in comparison with gender and age matched healthy schoolchildren, and looked for associations between PA and features of JIA.

**Methods:**

One hundred thirty-three patients, 7–20 years of age, participated. Disease activity, disability, functional ability, and pain were assessed and PA was measured by accelerometry through 7 days and compared to PA in age- and gender-matched healthy schoolchildren.

**Results:**

We found a significantly lower level of PA in patients compared to gender- and age-matched healthy schoolchildren both in average activity (counts per minute, cpm) (475.6 vs. 522.7, *p* = 0.0000018) and in minutes per day spent with cpm >1500 (67.9 vs. 76.4, *p* = 0.0000014), with cpm >2000 (moderate physical activity) (48.4 vs. 52.8, *p* = 0.0001, and with cpm >3000 (high physical activity) (24.7 vs. 26.5, *p* = 0.00015). A negative association (*β* = −0.213, *p* = 0.014) between active disease in weight bearing joints and high physical activity remained the only significant association between disease related factors and PA. Of the girls 19 % and of the boys 45 % (vs. 39 % and 61 % in the reference group) met standards set by Danish Health Authorities for daily PA in childhood.

**Conclusion:**

Children and adolescents with JIA are less physically active than their healthy peers and less active than recommended for general health by the Danish Health Authorities. This is not explained by pain or objective signs of inflammation. When inflammation has been curbed, restoration of an active healthy lifestyle should be highly prioritized.

## Background

Juvenile Idiopathic Arthritis (JIA) is the most common rheumatic disease of childhood with an annual incidence in the Western world of 16–150 per 100.000 children. During the last few decades, advances in medical treatment have changed the prognosis profoundly for these patients, and erosive arthritis resulting in functional disability during childhood is now rare. Spontaneous remission occurs, but 41–78 % of patients require continuous or recurrent treatment in adulthood, polyarthritis carrying the worst prognosis [1–4].

Rheumatoid Arthritis (RA) is epidemiologically associated with premature development of cardiovascular disease (CVD) and shortened lifespan. This has led to investigations of the vascular health in patients with JIA; indeed one finds arterial endothelial thickening, a preclinical sign of atherosclerosis that may develop into accelerated clinically important arteriosclerosis in adulthood [5–7]. Chronic inflammation with persisting systemic circulating inflammatory proteins could be a cause of vascular damage, but general physical inactivity in childhood is associated with well-known risk factors for premature development of cardiovascular disease [8] and could be an important contributor. Pain and fatigue are common complaints in patients with JIA and may well lead to an inactive lifestyle.

A sedentary lifestyle may also be harmful to the general development of the cardio-respiratory system and prevent a healthy development of the musculoskeletal system with the fine-tuning of motor skills, developing by usage. Moreover, an inactive lifestyle may be associated with social isolation, which may be a serious threat to normal mental development in childhood and adolescence.

Physical inactivity may thus impose an additional burden on the health of patients with JIA.

The primary aim of the present cross-sectional study was to assess the pattern of physical activity (PA) in children and adolescents with moderate to severe JIA, testing the hypothesis that patients with JIA are less physically active and possibly spend more time with sedentary activities than their healthy peers. Increased knowledge on this could be important for attention to exercise as part of the treatment for JIA. As self-reporting of physical activity (PA) is known to be unreliable [9, 10], we chose to monitor PA objectively by accelerometry.

Secondarily we wanted to look for a possible association between PA and disease activity, disability, pain, functional ability, JIA subgrouping, duration of symptoms, medication, and body mass.

## Methods

This is a population based descriptive cross-sectional study of objectively measured PA in patients with moderate to severe JIA, compared to age- and gender-matched healthy Danish schoolchildren.

The patients, markers of disease activity, and disability, signs of functional ability, and accelerometer measurement are described below in detail.

Data were collected throughout a period of two years from May 2011, covering different seasons and different times of the day for examinations. International recommendations for treatment of JIA remained unchanged during this period.

### Ethics

Ethics approval was obtained from The Committee on Biomedical Research Ethics in the Region of Sjaelland (SJ-220) and written information and accept obtained from patients or guardians according to the patients’ age.

### Patients

Children and adolescents with moderate to severe JIA, followed at a population based center for pediatric rheumatology, were invited to participate. All the patients with JIA living in the eastern part of Denmark (with a total population of 2.6 million) were seen at this clinic. Inclusion criteria were age between 7 and 20 years and JIA of a severity that necessitated treatment with Disease Modifying Medication (DMARD), either methotrexate (MTX) or biologics: TNF-α- or IL-6-inhibitors or T-cell co-stimulation inhibitor CTLA-4-Ig (Abatacept), or a need for frequently repeated intra-articular injections of corticosteroid. No one had other significant chronic conditions than JIA.

**Fig. 1:**
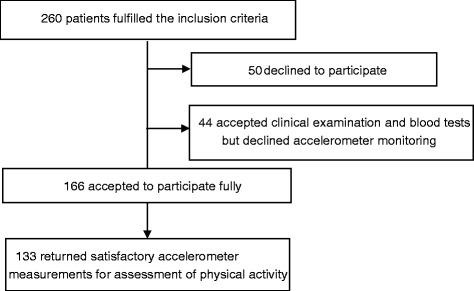
Number of patients entered in final analyses

Of 260 eligible patients, 166 accepted to participate in the whole investigation including monitoring with accelerometer, and of these, 133 patients returned accelerometer monitoring results of a sufficient quality for evaluation (Fig. 1).

The group of patients included in the final analyses (133 patients) is described by gender, age, weight, height, body mass index (BMI), JIA subgroup, treatment, and disease duration; number of patients in clinical remission with or without medication is specified (Table 1).

**Table 1:** Descriptive characteristics of the patients with valid accelerometer data

Total number	133
Females/males	99 (74 %)/34 (26 %)
Age (years)	Mean 14.0 (SD 3.37)
Height (m)	Mean 1.58 (SD 0.14)
Weight (kg)	Mean 51.2 (SD 15.2)
BMI (kg/m^2^)	Mean 20.1 (SD 3.93)
JIA subgroup:	
Systemic	4 (3 %)
Persisting Oligoarticular	38 (29 %)
Polyarticular, RF negative	41 (31 %)
Polyarticular, RF positive	3 (2 %)
Psoriasis associated	15 (11 %)
Enthesitis associated	31 (23 %)
Undifferentiated	1 (1 %)
Treatment:	
Methotrexate	31 (23 %)
Biologics	86 (65 %)
Only intra-articular injections	11 (8 %)
None	5 (4 %)
Disease duration^a^ in years	Mean 5.72 (SD 3.43)
Remission, still on medication	20 (15 %)
Remission, off medication	3 (2 %)

Summary statistics: Number with percentage in brackets, mean with SD in brackets, and median with range in brackets

*BMI* body mass index, *JIA* juvenile Idiopathic Arthritis, *RF* reumafactor

^a^Disease duration = duration of symptoms

### Reference data

Data on physical activity, measured by accelerometry, in healthy Danish schoolchildren, collected in The European Youth Heart Study (EYHS) [10, 11] and in The Copenhagen School Child Intervention Study (CoSCIS) [12] looking at risk factors for accelerated development of atherosclerosis, served as gender- and age-matched comparisons; in total 1696 measurements, 862 boys and 834 girls, all fulfilling standard criteria for validity given below. Every patient is compared to a group of healthy peers of same age +/− 1.5 years and of same gender.

Only data on Danish schoolchildren are used for comparison in the present study, as national cultural differences could be of importance.

The data on healthy schoolchildren have formed the basis for The Danish Health Authorities’ guidelines for daily PA in childhood [13].

### Clinical assessment of disease activity

All participants, diagnostically sub-grouped in accordance with ILAR criteria [14], were assessed clinically by a pediatrician experienced in pediatric rheumatology who did not take part in the investigation (Table 2). The participants were all seen as part of a routine control in the outpatient clinic; no one was seen because of acute disease flare. Actual disease activity was evaluated by a composite score, Juvenile Arthritis Disease Activity Score (JADAS) [15, 16], combining: *i)* number of joints with active inflammation (maximum 27 joints), *ii)* global disease scoring by physician, *iii)* global disease scoring by patient or parent according to age, and *iv)* truncated erythrocyte sedimentation rate (tESR). JADAS 27 is measured on a scale from 0 to 57.

**Table 2:** Markers of disease activity at the time of accelerometry

Number of active joints	Mean 1.29 (SD 2.64) (range 0–24)
Physician global VAS (0–10)	Mean 1.29 (SD 2.64) Median 1.0 (range 0–6.5)
Patient/parent global VAS (0–10)	Mean 1.6 (SD 2.0) Median 0.8 (range 0–8.2)
Truncated ESR (tESR) (mm/hour)	Mean 0,11 (SD 0,47)
High sensitivity CRP (mg/l)	Mean 1.92 (SD 3.86)
JADAS 27 (0–57)^a^	Mean 4.1 (SD 4.6) Median 3.1 (range 0–27)
Number of active weight bearing joints	Mean 0.9 (SD 1.3)
Tendinitis and/or entesitis (yes/no)	31 (23.3 %)
Patient Pain VAS (0–10)	Mean 2.9 (SD 2,7) Median 2.2 (range 0–10)
CHAQ (0–3)	Mean 0.4 (SD 0.5) Median 0.3 (ran

^a^JADAS 27 (0–57): a composite score of: number of joints with active disease (max 27), Physician global VAS, patient/parent global VAS and truncated ESR (_t_ESR)

In addition was noted the number of weight bearing joints with active inflammation. Also the occurrence of tendinitis and/or enthesitis was noted, as JADAS may have limited construct validity for psoriasis associated arthritis and enthesitis associated arthritis.

Global disease scoring was carried out by the physician on a Visual Analog Scale (VAS) from 0 (no sign of disease) to 10 (maximal disease burden) and the patient, or the parents according to age, marking on a VAS from 0 to10 how well the patient was doing considering having JIA, ten being worst. Remission was assessed according to Wallace criteria [17]. Disability was assessed by measuring limits of articular motion, and functional ability by the Danish version of Childhood Health Assessment Questionnaire (CHAQ) [18, 19]. In addition the child was asked to mark on a VAS (0–10) the level of pain during the last week, reflecting persistent pain.

Special charts were supplied for the clinical assessment. If the charts were insufficiently filled in, the missing information was found in the patient record for the actual visit in the outpatient clinic.

### Markers of inflammation

Besides the clinical assessment and measurement of hemoglobin, were measured numbers of neutrophils and thrombocytes, IgG, high sensitivity C-reactive protein (hsCRP) and erythrocyte sedimentation rate (ESR). ESR was truncated (tESR) according to the formula given by Consolaro et al. [15]: after converting ESR values of less than 20 mm/hour to 0 and ESR above 120 to 120, tESR was calculated as (ESR (mm/hour) − 20) / 10.

### Treatment

All patients or their parents were asked about the actual medication and the date for the last administration. The given information was in accordance with registered information.

### Anthropometry

Weight was measured with light clothing to the nearest 0.1 kg and height without shoes to the nearest 0.5 cm. BMI was calculated as weight/squared height.

### Accelerometry

An accelerometer, ActiGraph model GT1M (ActiGraph Inc., Pensacola, FL, USA), was used for measurement of volume and pattern of physical activity. The accelerometer measures accelerations in vertical movements with a filter for separating mere vibrations from movements. Accelerometers have now been used for more than a decade and have been found reliable for assessment of physical activity by comparison with energy expenditure [9]. The accelerometer collects data continuously, which in our investigation were set to be summed up in epochs of 5 s to capture the changing pattern of movement seen in children as compared to adults. The raw data were converted by the ActiGraph software to *i)* mean counts per minute (cpm), a measure of the total physical activity during total time of measurement, and *ii)* minutes per day spent on activity with more than 1500 cpm), *iii)* moderate physical activity (more than 2000 cpm, MPA), and *iv)* high physical activity (more than 3000 cpm, HPA), cut-points routinely used by others.

The accelerometer is a small, portable device fastened with an elastic band around the waist, the center of the body. In the study it was worn for seven days during all wake hours except when swimming or bathing, as the device is not waterproof. Time during the day spent without the device was noted in a small diary with information about the reason. Also time spent on horseback riding and cycling was noted, as accelerometry does not capture these physical activities accurately [20]. The accelerometer monitoring began the day after the clinical assessment in the outpatient clinic.

Adhering to the standards used in EYHS and CoSCIS for processing the accelerometer-data, only data collected during a minimum of 10 h per day for three days were considered reliable in assessing the physical activity. Periods of more than 10 min of zero count were considered as periods of “non-wear”.

### Statistical analyses

Summary statistics, mean and SD, median and range, or numbers with percentage, were chosen as descriptive measures.

The distribution of the level of physical activity in patients with JIA is compared to matched healthy schoolchildren by Related-Samples Wilcoxon Signed Rank Test, after log_10_ transformation because of a skewed distribution. Every patient is compared to a group of same age +/− 1.5 years and of same gender.

PA variables were log_10_ transformed because of a skewed distribution, and the dependence on disease related factors was tested by analysis of variance (one-way ANOVA), linear regression or by rank correlations/Spearman’s rho (non-parametric) or *t*-test as appropriate and specified.

Significance (p) is given as two-tailed and α selected as 0.05.

All analyses were done in SPSS19 or in Excel 2010.

## Results

The inclusion criteria were fulfilled by 260 patients, 50 patients refused to participate and 44 accepted clinical examination and blood tests but declined accelerometer monitoring. 166 patients accepted to participate fully but only 133 returned satisfactory accelerometer measurements for assessment of physical activity. Data are presented in Fig. 1.

Descriptive characteristics of the patients with valid accelerometer data are given in Table 1.

The group of eligible patients who chose not to participate (50 patients), or who returned no (44 patients) or unusable (33 patients) accelerometer data, were comparable to the group of fully participating patients (in total 133) with regards to distribution of gender, age, diagnostic subtype, and BMI. However, a significant difference (*p* = 0.001) was found in disease duration between the fully participating patients (mean 5.7 years, SD 3.5) and those not willing to wear the accelerometer (mean 7.3 years, SD 4.2). No markers of disease activity, described in Table 2, differed significantly between those participating fully in the program and those who returned unusable accelerometer data or only accepted blood tests and clinical examination.

Comparison of the distributions of the physical activity in patients with JIA and gender- and age-matched healthy schoolchildren by The Related-Samples Wilcoxon Signed Rank Test (2-tailed, α = 0.05) shows a significant difference both in average activity (cpm) and in the level of activity (minutes per day with cpm >1500 or >2000 (MPA) or >3000 (HPA)). Results are shown in Table 3.

**Table 3:** Physical Activity in patients with JIA compared to healthy schoolchildren

Physical Activity (PA)	Patients with JIA	Age- and gender-matched, healthy schoolchildren	*p*-value^a^
	133	In total 1692	
Mean cpm	475.6 (SD 178.8)	522.7	0.0000018
Minutes per day with cpm >1500	67.9 (SD 32.3)	76.4	0.0000014
Minutes per day with cpm >2000 (MPA)	48.4 (SD 24.7)	52.8	0.00010
Minutes per day with cpm >3000 (HPA)	24.7 (SD16.3)	26.5	0.00015

PA values were log_10_ transformed and pairwise compared by The Related-Samples Wilcoxon Signed Rank Test (2-tailed, *α* = 0.05)

^a^Comparison of the distributions of PA in patients with JIA and gender- and age-matched healthy schoolchildren

As seen in Table 4 only a relatively low percentage of participating patients fulfilled The Danish Health Authorities’ guidelines [13] for daily PA in childhood of more than 60 min a day of moderate physical activity (MPA) compared to healthy schoolchildren; in boys 45 % vs. 61 %, in girls 19 % vs. 39 %.

**Table 4:** Percentage of participating patients fulfilling The Danish Health Authorities’ guidelines [13] for daily physical activity (PA) in childhood of more than 60 min a day of moderate physical activity (MPA)

Percentage of boys with JIA/percentage of boys in the reference group: 45/61	
Percentage of girls with JIA/Percentage of girls in the reference group: 19/39	

A negative association (*β* = − 0.213, *p* = 0.014) was found between active disease in weight bearing joints and vigorous physical activity, while all other associations between disease related factors and PA were insignificant. In our group of patients 23 patients fulfilled the criteria for remission, 20 still on medication and three without. We found no difference in PA between patients in remission and patients with active disease. Details are given in Table 5.

**Table 5:** Disease characteristics in relation to level and pattern of physical activity

	Mean cpm	Minutes per day spent at >1500 cpm	Minutes per day spent at >3000 cpm (HPA)
Subgroup of JIA	*F* = 0.794	*F* = 0.927	*F* = 1.349
Analysis of variance	*p* = 0.531	*p* = 0.450	*p* = 0.255
Remission vs non-remission	*F* = 0.057	*F* = 0.369	*F* = 0.148
Analysis of variance	*p* = 0.811	*p* = 0.544	*p* = 0.701
Treatment^a^	*F* = 0.502	*F* = 1.127	*F* = 0.199
Analysis of variance	*p* = 0.607	*p* = 0.328	*p* = 0.820
Disease duration	*β* = 0.27	*β* = 0.005	*β* = 0.077
Linear regression	*p* = 0.758	*p* = 0.957	*p* = 0.376
Number of active joints	*β* = − 0.104	*β* = − 0.119	*β* = − 0.153
Linear regression	*p* = 0.235	*p* = 0.171	*p* = 0.078
Physician global VAS	rho = − 0.60	rho = − 0.062	rho = − 0.105
Spearman correlation	*p* = 0.489	*p* = 0.476	*p* = 0.228
Patient/parent global VAS	rho = − 0.072	rho = − 0.062	rho = − 0.018
Spearman correlation	*p* = 0.416	*p* = 0.487	*p* = 0.841
CRP	*β* = − 0.050	β = − 0.079	*β* = − 0.149
Linear regression	*p* = 0.591	*p* = 0.393	*p* = 0.107
JADAS 27	*β* = − 0.085	*β* = − 0.093	*β* = − 0.134
Linear regression	*p* = 0.340	*p* = 0.294	*p* = 0.144
Number of active weight-bearing joints	*β* = − 0.074	*β* = − 0.069	*β* = − 0.213
Linear regression	*p* = 0.400	*p* = 0.432	*p* = 0.014^b^
Number of joints with limited motion only	*β* = 0.054	*β* = 0.073	*β* = 0.105
Linear regression	*p* = 0.535	*p* = 0.405	*p* = 0.229
Tendinitis and/or enthesitis	473 vs. 455	68 vs. 63	27 vs. 26
*T*-test	*p* = 0.600	*p* = 0.402	*p* = 0.330
Pain VAS	rho = − 0.018	rho = − 0.002	rho = − 0.026
Spearman correlation	*p* = 0.841	*p* = 0.985	*p* = 0.775
CHAQ	rho = − 0.066	rho = − 0.065	rho = − 0.086
Spearman correlation	*p* = 0.457	*p* = 0.465	*p* = 0.334

^a^Treatment: either only intra-articular steroid injection or only methotrexate or biologicals with or without methotrexate

^b^Significance (p) is given as two-tailed and α selected as 0.05

In the subgroup “undifferentiated JIA” was only one patient and this patient had a relatively low PA; omitting this patient from the analysis of variance we found no significant association between JIA subgroup and PA.

## Discussion

We have found a significant difference, both in average PA (cpm) and in the level of PA, between children and adolescents with JIA and healthy schoolchildren (Table 3), supporting our hypothesis that patients with JIA are less physically active than their peers. We are not aware of other publications reporting objectively measured physical activity in children with JIA, but our findings are in accordance with a controlled cross sectional study by Lelieveld et al. [21] on physical activity in adolescents with JIA making use of self-reporting in an activity diary. Even though only 8 of the 30 included patients in that study had active disease, adolescents with JIA spent significantly longer time resting in bed and significantly shorter time on activities of moderate and high intensity; no association between PA and clinical disease activity or functional ability was found.

The difference in cpm and minutes per day spent on PA between healthy children and children with JIA (Table 3), may appear small, approximately 10 %; but an inactive lifestyle in a child with a chronic illness like JIA is likely to have a more permanent character than in a healthy child and thus be more harmful to normal physical development. Physical activity was not assessed in a recent study [22] reporting reduced aerobic capacity in patients with both active and inactive JIA but physical inactivity may have been a contributing factor. Unfortunately, we were not able to assess aerobic capacity, which is associated with PA, and presumably a parameter of higher prognostic value for general health related outcome in adulthood, than an episodic measurement of actual physical activity. It is noteworthy that only 61 % of boys and 39 % of girls in the reference group (Table 4) fulfilled the Danish Health Authorities’ guidelines for daily physical activity in childhood in maintaining general health. The small difference in PA between patients with JIA and healthy children found in our study may thus have more serious consequences than the figures suggest.

We used accelerometers in the assessment of PA in order to obtain more reliable data than mere self-reporting can yield. These motion sensors provide informative data on the complex movements of children and have been validated against methods for evaluating energy expenditure in the body [9]. The large investigations on healthy schoolchildren using accelerometers and well established international guidelines for interpretation of the data provide useful references according to age and gender. We asked the families to note time spent on cycling and horseback riding and swimming and we adjusted for swimming activity [23], but other activities were generally of a short duration and did not necessitate any corrections in the final assessment of the child’s physical activity. Reason given for not wearing the accelerometer was typically forgetfulness. We found only a modest association between self-reported PA and accelerometer measurements (data not shown).

PA is important for a healthy development and maintenance of all organs in the body as well as for acquiring and maintaining cognitive and social skills. In the wake of the decreasing physical demands in modern life style, the incidence of premature cardiovascular and metabolic diseases is rising internationally [24] but seem to begin leveling off in western countries presumably due to higher awareness of risk factors connected to lifestyle, and better medication. A large European study on physical activity in healthy schoolchildren (EYHS) showed a negative association between clustering of traditional risk factors for development of cardiovascular disease and levels of physical activity [8]. That investigation led to a general recommendation from the Danish Health Authorities [13] of a minimum of 1 h of moderate to vigorous PA per day, equivalent to more than >2000 cpm/day in our study, and corresponding to a walking speed of around 4 km/h. Only 19 % of the girls and 45 % of the boys in our study met the goal.

Physical inactivity in children and young adults with JIA may thus be an additional driver in the premature development in adulthood of atherosclerosis, which may be promoted by the chronic inflammation *per se*. Interestingly Aulie et al. [25] in a controlled study of vascular health in adults with persistently active JIA found an association between decreased augmentation index, a marker of arterial stiffness, and self-reported high physical activity, suggestive of a protective cardiovascular effect of high physical activity in patients with JIA. High BMI is another traditional risk factor for premature development of atherosclerosis, also associated with physical inactivity; the BMI of our patients was within the normal range.

The reduced level of PA in JIA is not easily explained. Except for an understandable negative association between vigorous physical activity and inflamed weight-bearing joints, we found no association between objective or subjective signs of disease activity, including remission, and level or pattern of physical activity. This is also in accordance with the findings by Lelieveld et al. [21].

Only three of our patients were in remission off medication. This may explain why the level and pattern of PA did not differ between those in remission and those with active disease. The finding raises concern, however, regarding a possible continuation of a lifestyle initiated in an active phase of a long lasting disease with no adjustments parallel to improvement.

We did not assess fatigue specifically. An association in patients with RA between higher level of physical activity and reduced level of fatigue has been described by Rongen et al. [26]. The fatigue had no relation to pain or disability, disease activity, cognition or coping, but fatigue could still be an inherent feature of inflammation, not easily captured in our evaluation of disease activity. Looking at patients with JIA, Ringold et al. [27] found a significant association between CHAQ, pain, and fatigue despite an overall low disease activity. In our study an association between PA and pain was not found. The pain scoring was, however, significantly higher than would be expected looking at the physician’s global disease scoring. This discrepancy has been addressed in several studies on pain in children with JIA [28–30].

Patients with JIA primarily need aggressive anti-inflammatory treatment in order to avoid bone erosion and destruction of joints, but when inflammation has been curbed, a return to a normal healthy lifestyle should be highly prioritized. As in the studies by Ringold et al. [27] and Lelieveld et al. [21], the disease activity in our patients was low, judged by objective signs and physician’s global VAS, and there is no reason to believe that mere intensification of medication would lead to higher level of PA.

Structured PA is added to conventional treatment of patients with JIA in several intervention studies [31–33]. A significant positive effect on general function by the end of the intervention was found in all studies. In the study by Takken et al. [32] an effect was only seen for the children with the poorest function at inclusion. In a Cochrane review, Takken et al. [34] found no significant support for the notion that adding PA to conventional medical treatment had a positive long-term effect on functional ability, quality of life, aerobic capacity or pain, although the reported short-term effects were promising. Long-lasting changes in a child’s lifestyle probably require a multidisciplinary approach built upon an idea of “self-efficacy” and addressing the complex and many-sided phenomenon of pain may be crucial. This must necessarily also involve the parents.

Importantly, Takken et al. in their review found no adverse effect of structured PA for children with JIA.

The strength of the present study is the relatively high number of participating children and adolescents with JIA with a well described disease not significantly different in disease characteristics from the rest of the patients with JIA living in the eastern part of Denmark. The reason given for not participating was not active disease but unwillingness to participate in yet another project during the course of the disease. This is also reflected in the significant difference in disease duration found between the fully participating patients and those not willing to wear the accelerometer.

The large reference material and the reliable objective way of assessing daily PA are excellent bases for our study.

In an investigation of a cross-sectional design only associations can be pointed out; causal pathways cannot be shown. We have not measured aerobic capacity; this is a weakness as aerobic capacity presumably is of greater prognostic value for continuing health than PA itself. We have not in our study assessed psychological or social factors, school attendance, disease beliefs, and beliefs regarding the benefits of PA, neither in the patients nor the parents. Understanding of these complex issues are of outmost importance for any kind of intervention in lifestyle.

We have not looked for special characteristics in those participants in the study able to live a physically and socially active life in spite of their chronic disease; such studies might provide further insight.

## Conclusion

The children and young adults with JIA who participated in our study are on average less physically active than their healthy peers, and less active than is recommended for general health by the Danish Health Authorities. This is not explained by objective, disease related factors and is thus not likely to be remedied by a more aggressive anti-inflammatory treatment. The well-known increased risk for cardiovascular and metabolic diseases related to an inactive lifestyle may thus reduce the prospect of a long healthy life without functional disability which our modern effective anti-inflammatory medicine offers. Incorporation in the medical treatment schedule of a program for regular physical exercise among peers may well benefit many of the patients. We speculate that we, by addressing the complex and multifaceted phenomenon of pain, may gain the patients’ attention also regarding positive changes in lifestyle.

## Abbreviations

BMI: Body mass index
CHAQ: Childhood Health Assessment Questionnaire
CoSCIS: The Copenhagen School Child Intervention Study
cpm: Counts per minute
CVD: Cardiovascular disease
DMARD: Disease Modifying Medication
ESR: Erythrocyte sedimentation rate
EYHS: The European Youth Heart Study
HPA: High physical activity
hsCRP: High sensitivity C-reactive protein
JADAS: Juvenile Arthritis Disease Activity Score
JIA: Juvenile Idiopathic Arthritis
MPA: Moderate physical activity
PA: Physical activity
RA: Rheumatoid arthritis
VAS: Visual Analog Scale
